# Editorial Notes

**Published:** 1918

**Authors:** 


### Editorial Notes.

**The Late Lieut.-Colonel Prof. Michell Clarke, Associate Editor.**

BY the death of Colonel Michell Clarke this *Journal* has lost one of its most valued contributors: the Editor mourns the loss of the senior member of his Editorial Staff. To a man of so many parts, whose reputation as a Consulting Physician was second to none in the West of England in his day and generation, his immense work for the *Journal,* continued over so many years, was relatively one of his minor interests. Yet it is significantly characteristic of Michell Clarke that his work for us was always carried out with punctilious care, his advice was ever sound, his guidance always to be trusted. In our obituary notice fuller justice is paid to the personal life and character and to the work of our friend and colleague; here we desire rather to express the feeling of irreparable loss to our readers of which the Editor is profoundly sensible. The first and dominant thought on hearing that Michell Clarke had passed from us was the untimeliness of death in the zenith of professional success and full vigour of life and faculty, the sense of work unfinished. Yet one glance at the bibliography of his contributions to Medicine serves to remind one what Clarke had accomplished, and that for such men the full harvest is never gathered. We understand that steps are being taken to perpetuate the memory of this great man, and we may feel assured that the Memorial will be worthy of the occasion; but we also trust that this opportunity will be used not merely to remind future generations of what Clarke was and did, but to carry on and develop his own contributions to Medical Science which he loved and served so well.

**Colonel J. A. Nixon.**

WARM congratulations to Colonel Nixon, A.M.S., appointed Consulting Physician to the Fourth Army in France.

**The case of Dr. John Henderson Bell.**

THE attention of all practitioners of medicine should be directed to the case of Dr. J. H. Bell, of Chelsea, who after practising for seventeen years, and by his upright life and professional skill had secured the confidence of a large number of patients, has been "convicted" apparently for an "act preparatory to an act" to enable a soldier to evade military service. A fairly complete account of the case has been reprinted, and has been sent to us by the Rev. Dr. Pentreath, Adderley Rectory, Market Drayton, Salop, and copies can be obtained by forwarding to him a stamped and addressed envelope. We urge all our readers to apply for a copy. The *Lancet* of March 2nd, in commenting on the case, states that "the manner in which the evidence was procured against him does not appear to us creditable to our times, and the quality of that evidence leaves the benefit of grave doubt on Dr. Bell's side. We cannot withhold from ourselves the conviction that we have here a case of miscarriage of justice." Briefly the accusation was that when called to see an Australian soldier (Orr), who he found with his leg resting on a table complaining of great pain, having as the soldier stated fallen off a bus the previous night, the doctor injected something in the knee to render the soldier unfit for military service. The examination seemed to cause great pain, so Dr. Bell said he gave an injection of morphine only, advising the patient to take a cab and go to a hospital. This was in January, 1917. In May, 1917, a trap was laid, Dr. Bell being called to see a "patient" complaining of nasal trouble. He proceeded to examine the man's nose, and painted the mucous membrane with supra-renal extract to get a better view of the nasal passages. Thereupon three persons concealed rushed into the room and arrested the doctor, the "patient" being Hawkins, a disguised warrant officer. Apparently it was assumed that the doctor was about to do something to the nose to render the "patient" unfit for service. But as it was admitted that the doctor had no instruments which would have any such effect, it is difficult to understand how such assumptions could support the case. Several well-known surgeons and nasal specialists have considered the evidence, and certify as follows :—

### Medical Statements.

We certify that we have individually considered the evidence in the case of Dr. John Henderson Bell.

We are emphatically of opinion :—

(1) That Dr. Bell's examination of Orr was proper, and his advice that he should go to a Military Hospital correct.

(2) That, in view of the presence of swelling of the knee and the man's complaint of great pain, the injection of a quarter of a grain of morphia was justifiable.

(3) That, as the leg was exposed, there was nothing unusual in the injection being made into the thigh.

(4) That if any fluid capable of causing incapacity for Military Service had been injected into the knee-joint, the man would not have been able to walk about as he did during the subsequent three days before going to hospital.

(Sgd.) WILLIAM HY. BATTLE, F.R.C.S.,
Senior Surgeon St. Thomas' Hospital, &c.

(Sgd.) WALTER G. SPENCER, F.R.C.S.,
Senior Surgeon Westminster Hospital, &c.

(Sgd.) EDRID M. CORNER, F.R.C.S.
Surgeon to St. Thomas' Hospital, the King George Hospital, the 5th London General Hospital, &c.

WE CERTIFY that we have individually considered the evidence in the case of DR. JOHN HENDERSON BELL.

WE are emphatically of opinion :—

(1) That Dr. Bell's examination of the nasal passages in the case of the man Hawkins was in every way usual and proper.

(2) That with the instruments Dr. Bell had with him it would have been impossible for him to have done anything injurious to the nasal passages.

(3) That nothing in Dr. Bell's examination could possibly be regarded as preliminary to, or leading up to, the committing of a criminal act.

(Sgd.) ST. CLAIR THOMSON,
M.D., F.R.C.P. Lond., F.R.C.S. Eng.

(Sgd.) SYDNEY R. SCOTT,
M.S., M.B. Lond., F.R.C.S. Eng.

(Sgd.) FRANK A. ROSE,
M.A., M.B. Cantab., F.R.C.S. England.

The Australian soldier was Orr, who was arrested for desertion while in the hospital to which Dr. Bell referred him, and only then accused Dr. Bell of having injected something into his leg to make him unfit for service. But there was no evidence of such an injection having been made, while as for the "bogus" nose case, everyone who examines the nose of any patient ought to do much the same as Dr. Bell was proved to have done. It has never been shown that Dr. Bell received any monies in connection with the matter beyond one guinea for his visit to Orr, and which was duly entered in his books.

An appeal to the Home Secretary for re-trial was refused, on the ground that no fresh evidence was adduced, but what possibility is there of such fresh evidence being adduced by Dr. Bell!

If guilty Dr. Bell has prostituted his profession by acts which merit the contempt of his fellows; but—if innocent! the cruelty of such a miscarriage of justice is scarcely measurable. We do not suggest that either magistrates or his judge have been impelled by any but the most honourable of motives, but it is obvious enough to his medical colleagues that the lay mind might be misled by surgical procedures which were innocent and quite usual methods of investigation.

It cannot be doubted that the General Medical Council will go thoroughly and carefully into the whole case, and, *exercising their judicial powers*, deal with it only on its merits. To the Council we look for guidance, either for the protection of society from a scoundrel or the vindication of a martyr, whichever Dr. Bell may be; thus on their judgment hangs an issue of big moment, and for each member a great responsibility.

Further, we protest that to invade the consulting room with a bogus patient, who, in order to make evidence in a trial, is instructed to complain of imaginary symptoms, and then called on to give, as evidence, his layman's views of *what he imagines the doctor intended to do*, places every medical practitioner in a dangerous position, which ought to be illegal, for it certainly is unfair and unbearable.

